# Single-cell RNA sequencing reveals the Müller subtypes and inner blood–retinal barrier regulatory network in early diabetic retinopathy

**DOI:** 10.3389/fnmol.2022.1048634

**Published:** 2022-12-01

**Authors:** Yan Wang, Xiongyi Yang, Qiumo Li, Yuxi Zhang, Lin Chen, Libing Hong, Zhuohang Xie, Siyu Yang, Xiaoqing Deng, Mingzhe Cao, Guoguo Yi, Min Fu

**Affiliations:** ^1^Department of Ophthalmology, South China Hospital of Shenzhen University, Shenzhen, China; ^2^The Second Clinical School, Southern Medical University, Guangzhou, Guangdong, China; ^3^Department of Anesthesiology, Shenzhen Hospital, Southern Medical University, Shenzhen, Guangdong, China; ^4^Department of Ophthalmology, The Seventh Affiliated Hospital, Sun Yat-Sen University, Shenzhen, China; ^5^Department of Ophthalmology, The Sixth Affiliated Hospital, Sun Yat-Sen University, Guangzhou, Guangdong, China; ^6^Department of Ophthalmology, Zhujiang Hospital, Southern Medical University, Guangzhou, Guangdong, China

**Keywords:** single-cell RNA sequencing, blood–retinal barrier, Müller cell, diabetic retinopathy, communication network

## Abstract

As the basic pathological changes of diabetic retinopathy (DR), the destruction of the blood-retina barrier (BRB) and vascular leakage have attracted extensive attention. Without timely intervention, BRB damage will eventually lead to serious visual impairment. However, due to the delicate structure and complex function of the BRB, the mechanism underlying damage to the BRB in DR has not been fully clarified. Here, we used single-cell RNA sequencing (RNA-seq) technology to analyze 35,910 cells from the retina of healthy and streptozotocin (STZ)-induced diabetic rats, focusing on the degeneration of the main cells constituting the rat BRB in DR and the new definition of two subpopulations of Müller cells at the cell level, *Ctxn3*^+^Müller and *Ctxn3*^−^Müller cells. We analyzed the characteristics and significant differences between the two groups of Müller cells and emphasized the importance of the *Ctxn3*^+^Müller subgroup in diseases. In endothelial cells, we found possible mechanisms of self-protection and adhesion and recruitment to pericytes. In addition, we constructed a communication network between endothelial cells, pericytes, and Müller subsets and clarified the complex regulatory relationship between cells. In summary, we constructed an atlas of the iBRB in the early stage of DR and elucidate the degeneration of its constituent cells and Müller cells and the regulatory relationship between them, providing a series of potential targets for the early treatment of DR.

## Introduction

Due to the continuous increase in the global prevalence of diabetes, diabetic retinopathy (DR) is still the main cause of vision loss in many developed countries (Antonetti et al., 2021). Among 246 million patients with diabetes, approximately one-third have signs of diabetic retinopathy (Teo et al., 2021). The pathogenesis of DR is very complex and has not yet been fully elucidated, and its basic pathological changes are the destruction of the blood-retina barrier (BRB) and the formation of retinal neovascularization (Klaassen et al., 2013).

The BRB is composed of the inner BRB (iBRB) and the outer BRB (oBRB), which regulate the movement of liquids and molecules between the ocular vascular bed and retinal tissue and prevent macromolecules and other potentially harmful substances from penetrating the retina (Guymer et al., 2004). The iBRB is established by the tight junction between endothelial cells (Fresta et al., 2020), which is located on the basal layer covered by Müller cells, while pericytes are wrapped in the basal layer and in close contact with endothelial cells. Therefore, damage to the iBRB is considered to be one of the main causes of retinal vascular diseases and has also attracted increasing research and attention. Previous studies have shown that retinal Müller cells play a key role in maintaining the structure and characteristics of the iBRB by relying on their own structural characteristics and strong secretion (Abbott et al., 2006). Pericytes not only provide mechanical support but also form a barrier between microvessels and tissue spaces with endothelial cells, interact with endothelial cells through physical contact and paracrine signals, and regulate the permeability of the BRB barrier, retinal blood flow, and stress response, which are important factors to maintain the stability of the internal environment (Fresta et al., 2020). Most of the current studies focus on the middle and late stages of DR, but more and more evidence supports that the inflammatory response of the retina in the early stage precedes microvascular disease. In the mouse model, the increase of inflammatory mediators such as MIP-1, IL-1, and IL-3 precedes the formation of new blood vessels, and is considered to be the cause of retinal nerve cell death in early diabetes. Studies on some DR animal models have confirmed that inhibiting or knocking out proinflammatory molecules can inhibit diabetes-induced retinal vascular and neurodegenerative diseases. Due to the large number of cells involved in the inflammatory response and the complex regulatory mechanism between cells, the mechanism of iBRB injury in the early stage of DR is not completely clear, and its exploration is still a major challenge.

To solve the above problems, researchers have used single-cell RNA sequencing (scRNA-seq) technology. We used five rat retinal samples (2 normal SD rats, 1 DR sample at 2 weeks, 4 weeks, and 8 weeks each), constructed a single-cell map of a total of 35,910 transcripts, and explored the cellular mechanism of iBRB injury in the early stage of DR. Single-cell sequencing can better reveal the structure of the iBRB and the interaction between iBRB cells through high-resolution detection of each cell in the retina.

We explored the characteristics of pericytes, endothelial cells and two Müller cell subtypes, and constructed a regulatory network among them in the early stage of DR. Our research once again emphasizes the important role of the iBRB and provides a new target for protecting the iBRB and inhibiting the further development of DR, which has far-reaching clinical significance and social value.

## Results

### An atlas of cell types in the inner blood–retinal barrier

**Figure 1:**
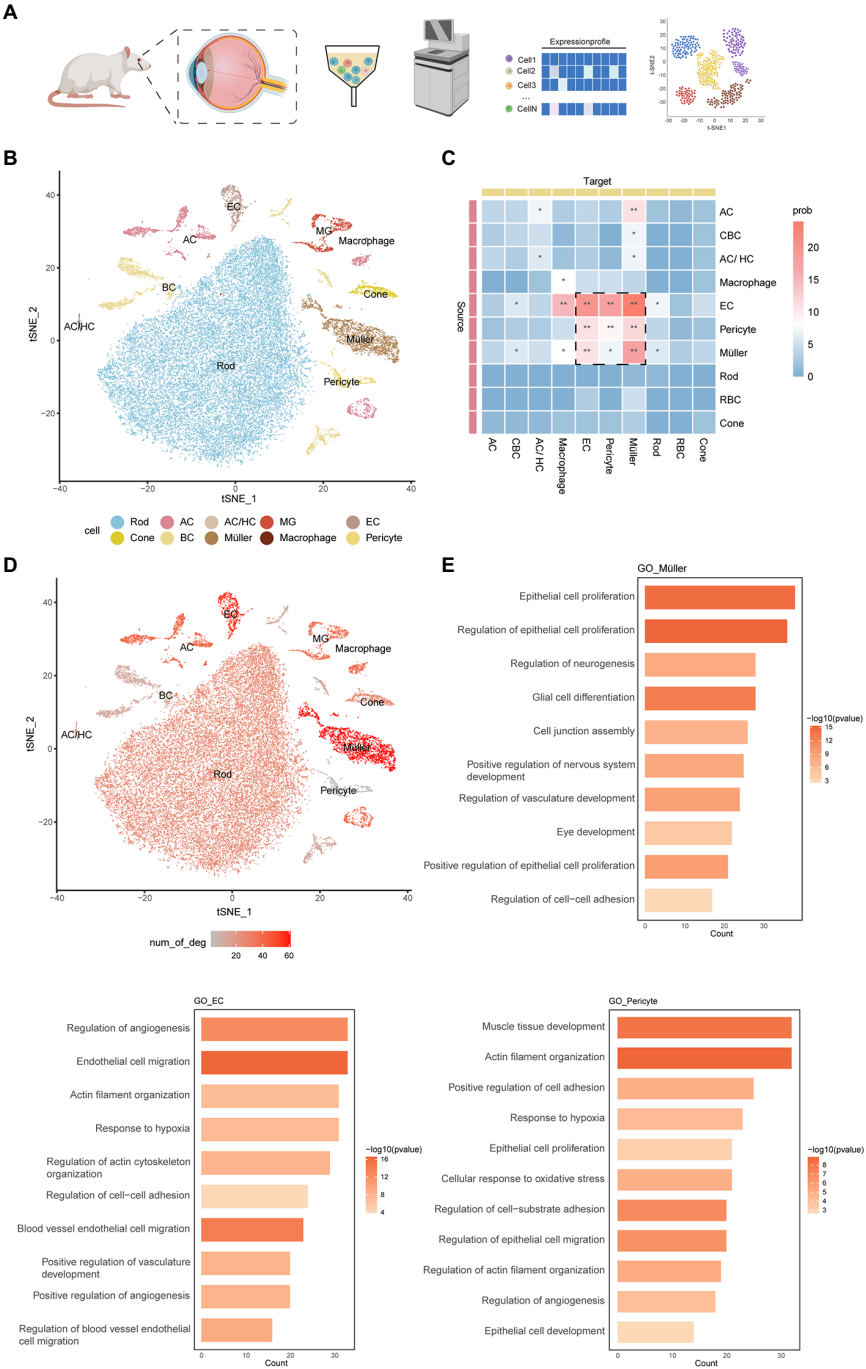
An atlas of cell types in the inner blood–retinal barrier. **(A)** Experimental design overview. Cell suspensions were collected from the experimental group (2 weeks, 4 weeks, 8 weeks) and the control group and subjected to 10x library preparation and sequencing, followed by downstream analyses. **(B)** tSNE plot showing different cell types. Cells assigned to the same cluster are similarly colored. AC, amacrine cell; HC, horizontal cell; EC, endothelial cell; MG, microglial cell; BC, bipolar cell. **(C)** The heatmap shows the communication relationships between cells. The source is the sender of the signal, the target is the receiver, and more asterisks indicate stronger communication. Strong communication is shown in red, and weak communication is shown in blue. Prob represents the strength of intercellular communication. **(D)** The feature plot shows the number of DEGs in different cells under DR and normal conditions. The three cells with the largest numbers were pericytes, Müller cells, and endothelial cells. **(E)** The histogram shows the GO enrichment results of pericytes, Müller cells, and endothelial cells, with length representing count and color representing -log10 (*p*-value).

To simulate early DR, we used streptozotocin (STZ) to induce type 2 diabetes in rats. We stripped the retinas of 5 rats (2 normal rats and 3 treated with STZ for 2, 4, and 8 weeks) and isolated and diluted these samples for single-cell transcriptome sequencing (Figure 1A). After quality control, 35,910 high-quality cells were retained: 11,073 cells from normal samples and 24,837 cells from diabetic rats (Supplementary Figure S1A). We performed unbiased clustering on cells with similar gene expression profiles and found 34 clusters. A red blood cell type defined as having a very small number of cells was removed, so we finally obtained 33 cell subtypes. We used uniform manifold approximation and projection (UMAP) dimensional reduction to visualize all cell subtypes. According to a previously reported list of genes expressed in retinal cell types (Supplementary Table S1), we divided 33 clusters into 10 cell types, including a blood-derived macrophage type and a retinal resident cell type. Resident cells included neuronal cells (rod cells, cone cells, horizontal cells, amacrine cells, and bipolar cells), glial cells (Müller cells and microglia cells), endothelial cells, and pericytes (Figure 1B; Supplementary Figures S1B–D).

Based on CellChat analysis, we found that endothelial cells, pericytes, and Müller cells have more complex connections and communication modes than other cells (Figure 1C). These cells are involved in the composition and regulation of the blood–retinal barrier. In addition, we counted the differentially expressed genes (DEGs) of various retinal cells in normal rats and diabetic rats and found that the main cells that make up the iBRB—Müller cells, endothelial cells, and pericytes—changed significantly in the early stage of DR (Figure 1D). Gene Ontology (GO) enrichment analysis showed that all three cell types were related to the formation and proliferation of blood vessels, hypoxia, and cell adhesion, pericytes were also related to vasoconstriction, and Müller cells were also related to the formation and development of neurons (Figure 1E). Therefore, we next focused on the cellular and molecular mechanisms involved in the iBRB microvascular regulatory network, and we also newly defined the marker gene of these cells (Supplementary Table S2).

### The characteristics of the two subtypes of Müller cells

**Figure 2:**
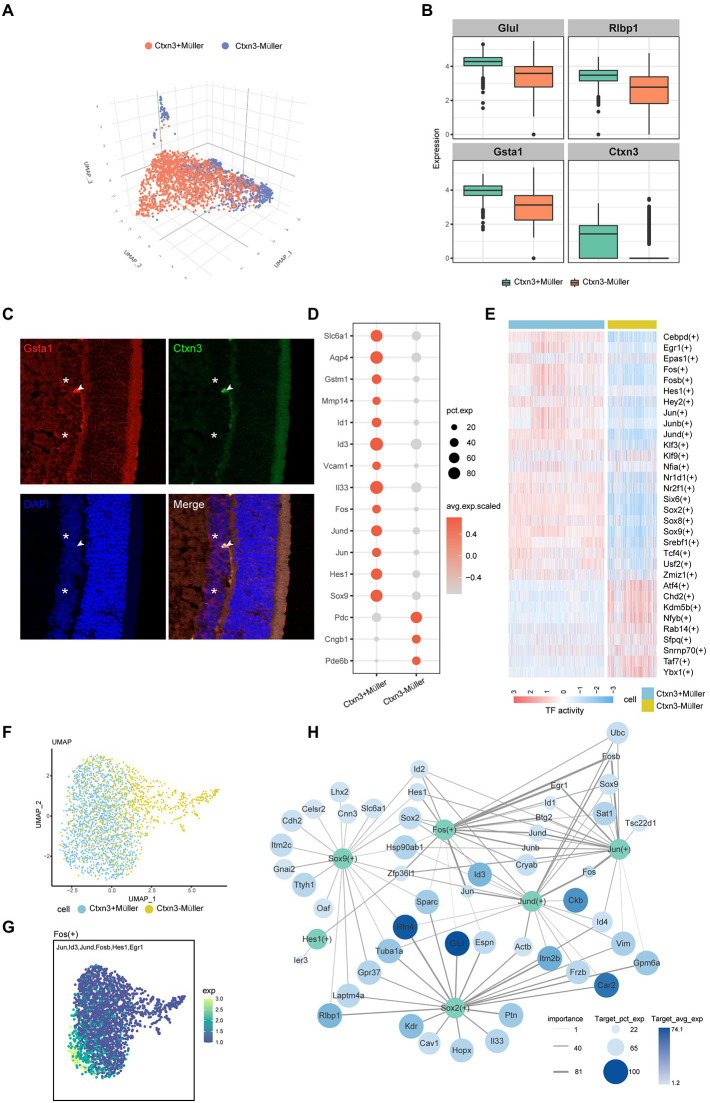
The characteristics of the two subtypes of Müller cells. **(A)** Three-dimensional UMAP plot of *Ctxn3*^+^Müller and *Ctxn3*^−^Müller cells. The different colors correspond to different cell types. **(B)** Box diagram showing the expression of the marker genes, which distinguishes the two groups of Müller cells. **(C)** Immunofluorescence labeling for *Gsta1* (red) and *Ctxn3* (green) and DAPI nuclear staining (blue) in the rat retina. *Ctxn3*^+^Müller are indicated by arrowheads, and *Ctxn3*^−^Müller are indicated by asterisk (40×, scale bar 20 μm). **(D)** The bubble plot shows the expression of important cytokines in *Ctxn3*^+^Müller and *Ctxn3*^−^Müller cells. The bubble size represents pct.exp., and the color represents avg.exp.scaled. **(E)** Heatmap showing the activity of important TFs in *Ctxn3*^+^Müller and *Ctxn3*^−^Müller cells according to AUCell. **(F)** UMAP plot showing two subclusters of Müller cells. **(G)** UMAP showing the expression of target genes of Fos. The color from dark to light represents increased expression. **(H)** The network diagram shows the important TFs in *Ctxn3*^+^Müller cells and their downstream target gene associations. TFs are shown in green, and the color of the target gene represents Target_avg_exp; the size of the circle represents Target_pct_exp, and the line thickness represents importance. TF with avg. exp. > 6 among the top 10 avg. exp. * pct.exp. values was selected as the research object. We selected the top 20 genes by the Target_avg_exp*importance value among the target genes with importance >5 for each TF.

Müller glial cells are the most important glial cells in the rat retina. They can provide energy and a variety of neurotrophic factors for nerve cells, participate in the transport of neurotransmitters and ions, and play an important role in maintaining the morphological structure of the BRB, protecting the integrity of neurons, and maintaining the homeostasis of the retinal environment. In the early stage of DR, Müller cell injury can lead to the destruction of retinal homeostasis. According to the previous markers (*Apoe*, *Glul*, and *Clu*), we identified two groups of Müller cells and named the two groups of cells *Ctxn3*^+^Müller and *Ctxn3*^−^Müller (Figures 2A,B), according to the expression of the *Ctxn3* gene. We use immunofluorescence experiment to explore the existence of two subpopulations, but more experiments need to be further implemented due to the ambiguity of the results (Figure 2C; Supplementary Figure S2A).

By analyzing the differentially expressed genes of the two groups of Müller cells (Figure 2D), we identified γ-aminobutyric acid (GABA) transporters, as well as the ability to remove excess glutamate through glutamate transporters on the cell membrane and intracellular glutamine synthetase to avoid cytotoxicity caused by glutamate accumulation(Coughlin et al., 2017). The ingested GABA is then acted on by GABA aminotransferase on mitochondria, and *Ctxn3*^−^Müller cells showed lower levels of this transporter than *Ctxn3*^+^Müller cells. We also found that both types of Müller cells express glutathione transferases (GSTs) and aquaporins (AQP4). GSTs protect retinal neurons from oxidative damage. AQP4 is involved in the drainage process of the neuroretina and avoids retinal tissue edema. Interestingly, glutathione transferase and AQP4 are more highly expressed in *Ctxn3*^+^Müller cells. In addition, the overexpression of matrix metalloproteinase (MMP-14) and inhibitor of DNA binding/differentiation (ID) were observed in *Ctxn3*^+^Müller cells. The former participates in the degradation of collagen in the extracellular matrix, which has an important protective effect on the fibrosis of retinal tissue after ischemia and hypoxia. Moreover, MMP-14 and Id proteins were related to neocapillaries. In terms of inflammation, *Ctxn3*^+^Müller cells specifically overexpressed the vascular adhesion molecule VCAM1, which may suggest its possible role in the pathogenesis of diabetic microangiopathy. Furthermore, Müller cells can release inflammatory factors such as interleukin 33 (Il33), suggesting that *Ctxn3*^+^Müller cells can participate in the inflammatory response in the retina. Interestingly, however, many genes related to photosensitivity, such as *Pde6b* and *Cngb1*, were observed to be highly expressed in some *Ctxn3*^−^Müller cells. Combined with previous studies, Müller cells may exist as a special type of optical fiber while researchers have found that Müller cells also have an inseparable relationship with photoreceptor differentiation. However, our data cannot avoid the possibility of photoreceptor containment and whether *Ctxn3*^−^Müller cells have these effects here is our further research content (Reichenbach and Bringmann, 2013).

To further explore the role of *Ctxn3*^+^Müller cells in disease, we conducted SCENIC analysis (Figure 2E). Two types of transcription factors are mainly upregulated in *Ctxn3*^+^Müller cells: (1) AP-1 family members, such as Jun, Fos, and Jund; and (2) SOX family members, such as Sox2 and Sox9. We showed the expression of target genes downstream of important transcription factors with UMAP plots (Figures 2F,G; Supplementary Figure S2B). Through the network diagram, we found that the AP-1 family can regulate the changes in Id protein (Figure 2H). Id1-4 have been observed to be upregulated in *Ctxn3*^+^Müller cells. Id1 and Id3 were closely related to angiogenesis in previous studies and are loops in the amplification loop of vascular endothelial growth factor (Vegf) and transforming growth factor Tgf-β (Song et al., 2011; Georgiadou et al., 2014; Young et al., 2015). Similarly, elevated levels of *Egr1*, a gene downstream of Fos and Jun, have been shown to mediate retinal vascular dysfunction in diabetic rats (Ao et al., 2019). The above findings suggest that *Ctxn3*^+^Müller cells actively participate in the occurrence and development of the disease. Sox proteins are a class of transcription factors found in animals. These molecules belong to the DNA binding proteins of the high mobility group (HMG) superfamily. We observed that Sox2 and Sox9 jointly regulate many genes related to Müller cell structure and function, such as retinol-binding protein 1 (*Rlbp1*), receptor *Gpr37,* and integrated membrane protein. Many studies have shown that Notch signaling is an important regulator of developmental and postdevelopmental processes. *Notch2* and the Notch downstream genes *Hes1* and *Sox9*, which are abundant in mature retina, are important factors for retinal Müller cells to maintain structural stability and development and survival (Muto et al., 2009; Bhatia et al., 2011; Zhu et al., 2013; Bachleda et al., 2016). Notably, Sox2 can also regulate the release of Il33, which further proves that *Ctxn3*^+^Müller is involved in retinal inflammation. In conclusion, we believe that *Ctxn3*^+^Müller cells, as an important component of Müller cells in the iBRB regulatory network, play a major role by expressing angiogenic factors and coordinating angiogenesis-related transcription factors. We found potential targets that may regulate and affect the participation of *Ctxn3*^+^Müller cells in destruction of vascular function, angiogenesis, and the inflammatory response. We also explored important factors that maintain the stability of cell structure and function, providing new ideas for the treatment of diseases.

### Characteristics and interrelationship of endothelial cells and pericytes

**Figure 3:**
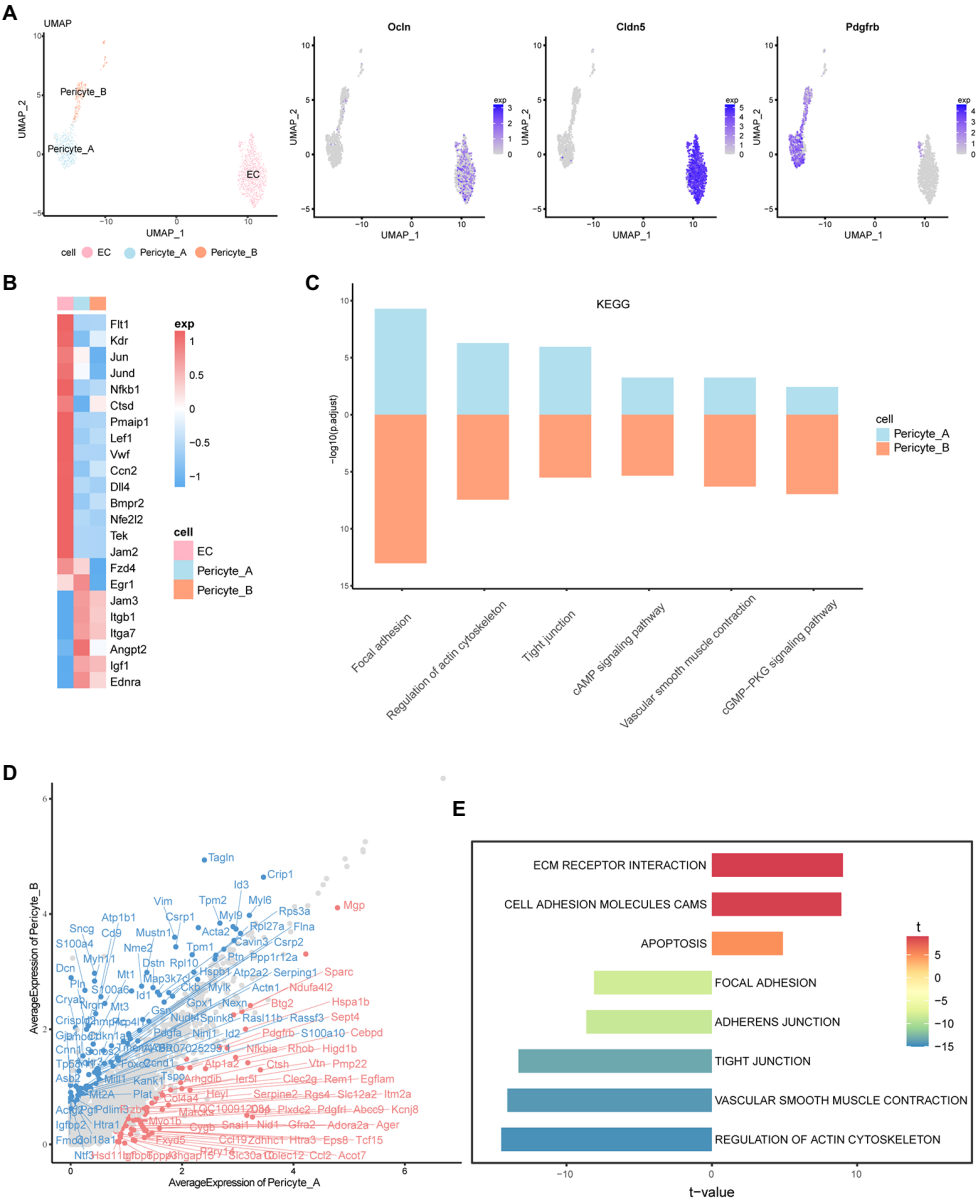
Characteristics and interrelationship of endothelial cells and pericytes. **(A)** UMAP diagram of EC, Pericyte_A, and Pericyte_B. The feature plot shows the expression of marker genes in each of the three cells. **(B)** The heatmap shows the expression of important cytokines in the three groups of cells. High expression is in red, and low expression is in blue. **(C)** The histogram shows the KEGG enrichment results of Pericyte_A and Pericyte_B, and the length represents -log10 (p.adjust). **(D)** The scatter plot shows the difference in gene expression between the two groups of cells. The red one is the upregulated gene of Pericyte_A relative to Pericyte_B. Genes with abs (avg_logFC) ≥ log2 (1.55) and p_val_adj ≤0.01 are labeled. **(E)** The two-way histogram shows the GSVA enrichment results of Pericyte_A compared with Pericyte_B, and both length and color represent the t value. T value is the statistical value of the t test, which is used to infer the size of *p* value.

Cluster 8 was defined as the endothelial cell population because of the high expression of claudin-5 (*Cldn5*) and occludin (*Ocln*). Claudin-5 is the main structural determinant of the paracellular endothelial barrier, which can promote the closure of tight junctions, thereby reducing vascular permeability and enhancing endothelial barrier function. Occludin is also a transmembrane component of tight junctions between the endothelium, which can regulate the permeability of the BRB. Platelet-derived growth factor receptor beta (pdgfrβ), which plays essential roles in the development of vascular mural cells, including pericytes and vascular smooth muscle cells, was highly expressed in clusters 20 and 27. Therefore, we defined these two cell groups as pericytes, named Pericyte_A and Pericyte_B, respectively (Figure 3A).

By analyzing the characteristics of vascular endothelial cells, we found a series of important factors that may be new targets for future disease treatment (Figure 3B; Supplementary Figure S3A). In addition to the characteristic markers of endothelial cells and Vegf receptors, we observed that in DR, the expression of the mitogen-activated protein kinase ERK and its downstream AP-1 family, NF-κB, and other factors in endothelial cells was high. NF-κB further promoted the expression of *Gadd45g*, which is related to cell growth and the apoptosis-related genes *Ctsd* and *Pmaip1*, and regulated the survival of endothelial cells. In addition, endothelial cells highly express the Wnt receptor Fzd4 and the transcription factor lymphoid enhancer Lef1. The typical Wnt/β-catenin signaling pathway has been reported to be one of the key systems that coordinates endothelial cell behavior and regulates angiogenesis(De, 2011), and Lef1 is mainly involved in this signaling pathway. Activation of the classical pathway further promotes the transcription of AP-1 family genes, such as *Jun,* downstream and regulates cell function. There are many factors that affect endothelial injury and promote angiogenesis. In addition to the important role of Vegf and angiotensin, more studies have shown that early growth factor 1 (*Egr1*) is the main mediator in response to Vegf stimulation (Mechtcheriakova et al., 1999). In our data, Vegf promotes an increase in *Egr1* content, which may affect the proliferation and differentiation of endothelial cells and promote angiogenesis and injury. Moreover, we found that von Willebrand factor (VWF) is highly expressed in endothelial cells and is an important factor that aggravates vascular endothelial cell damage and promotes the development of retinopathy (Lip et al., 2000). In addition, endothelial cells specifically overexpress connective tissue growth factor CTGF (*Ccn2*), which is related to the pathogenesis of microvascular complications in diabetes (Tikellis et al., 2004). More studies have shown that hyperglycemia increases the expression of CTGF in rat and human retinas, accompanied by an increase in Vegf (Hughes et al., 2007) and Tgf-β (Tikellis et al., 2004) expression, suggesting that CTGF may be an important target for the treatment of DR. In conclusion, through the analysis of specific factors in endothelial cells, we revealed its role in disease, providing new targets for slowing down the damage to the iBRB.

Interestingly, in the early stage, endothelial cells seem to have protective mechanisms to reduce their own damage. We found that the Notch pathway is significantly activated, and studies have shown that the Vegf and Notch signaling pathways are involved in the process of inducing and selecting tip cells. Inhibiting the Notch pathway can cause the proliferation of vascular endothelial cells and promote the formation of neovascular branches (Hellstrom et al., 2007). The increase in the content of the *Dll4* gene downstream of Vegf can inhibit the formation of new branches and neovascularization of blood vessels through the Notch pathway. Furthermore, endothelial cells increase the contents of bone morphogenetic protein receptor (Bmpr2) and nuclear factor erythrocyte-related factor 2 (Nfe2l2). The lack of the former is a factor leading to severe endothelial inflammation (Simic, 2013), while Nfe2l2 can induce the expression of the glutathione transferase GST gene (Suzuki and Yamamoto, 2015), which can protect cells from damage by active substances. Therefore, we suspect that endothelial cells may play a role in self-protection in the early stage of disease through the above mechanisms. In addition, the tyrosine kinase receptor Tie2, which plays a central role in vascular stability, is mainly located on vascular endothelial cells, and it can be activated by angiopoietin 1 (Angpt1) secreted by Pericyte_A. Activated Tie2 can enhance the survival, adhesion, and integrity of endothelial cells to stabilize blood vessels (Campochiaro and Peters, 2016).

As one of the main cellular components of retinal microvessels, pericytes are closely related to endothelial cells. KEGG analysis showed that Pericyte_A and Pericyte_B showed enhanced vascular smooth muscle contraction, cGMP-PKG, cAMP, and other signaling pathways, which are closely related to the characteristics of pericytes such as regulation of the blood flow of local microvessels through contraction (Figure 3C). In addition, both groups of pericytes have increased tight junctions and other signaling pathways, which are closely related to the location and morphology of pericytes. Pericytes are in close contact with capillary endothelial cells, connecting and adhering to each other. Tight junctions formed by Jamb (Jam2)-Jamc (Jam3) and integrins highly expressed on the surface of pericytes play an important role in maintaining the iBRB and mediating the adhesion of endothelial cells and pericytes.

Next, we compared the two groups of pericytes (Figure 3D; Supplementary Figure S3B). Interestingly, we found that in addition to Pdgfa, Pericyte_ B also expressed Pgf and Ntf3. Pgf was found to negatively regulate endothelial cell barrier function through Vegfr1. Pgf and its receptor Vegfr1 may be new therapeutic targets for angiogenic diseases in human retinal endothelial cells in a high glucose environment. The neurotrophic factor Ntf3 can promote the development and survival of retinal neurons. In contrast, Pericyte_A secretes angiopoietin 2 (*Angpt2*). In ischemic diseases such as DR, the upregulation of Angpt2 inactivates Tie2, leading to vascular leakage, pericyte loss, and inflammation. The coexpression of Angpt2 and Vegfa will accelerate neovascularization in developing retina and ischemic retina models (Campochiaro and Peters, 2016). Both groups of cells express insulin-like growth factor (*Igf1*), and the overexpression of *Igf1* may lead to the accumulation of Vegf. These changes increase the permeability of blood vessels and are related to the loss of vascular tight junction integrity (Chantelau et al., 2008). In terms of receptor expression, both groups of pericytes express endothelin receptor (ETA), reflecting the contractile characteristics of pericytes themselves. In addition, Pericyte_A showed significantly upregulated Pdgfrb and advanced glycation end product-specific receptor (Ager), indicating that it may have a stronger ability to migrate to and recruit endothelial cells and may be more susceptible to the effects of high glucose(Zhang et al., 2020).

Gene set variation analysis (GSVA) showed that Pericyte_A had increased ECM receptor interactions and other pathways, while vascular smooth muscle contraction and actin cytoskeleton regulation and other pathways were significantly enriched in Pericyte_B (Figure 3E). Combined with the GSVA and the expression characteristics of factors and receptors secreted by the two groups of peripheral cells, we found that they play different roles in iBRB regulation: Pericyte_A seems to be more vulnerable to a hyperglycemic environment and may be more vulnerable to damage and loss, while Pericyte_B is more similar to vascular smooth muscle cells and has a stronger ability to mediate peripheral cell contraction and regulate retinal blood flow; it also secretes a variety of factors to regulate other cells in the iBRB.

### Communication network between the Müller and iBRB

**Figure 4:**
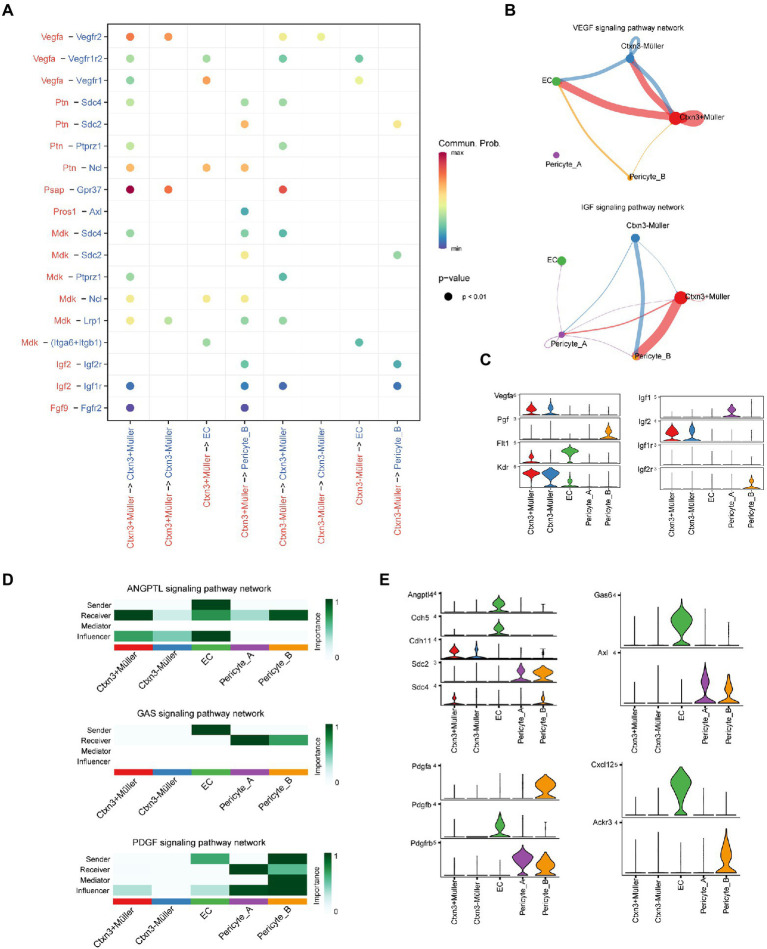
Communication network between Müller cells and the iBRB. **(A)** Comparison of the significant ligand–receptor pairs. The color indicates the value of communication probability, and the point size indicates the p-value. Ligands and signal senders are marked in red, while receptors and receivers of signals are marked in blue. **(B)** VEGF and IGF signaling pathway networks among Müller cells, ECs, and pericytes. The thickness of the line represents the strength of the communication signal. **(C)** Violin diagram shows the expression of genes of ligand–receptor pairs in the VEGF and IGF signaling pathway network. **(D)** The heatmap shows the sender, receiver, mediator, and influencer of each signaling pathway network. The color represents the strength of the communication signal. **(E)** Violin diagram showing the expression of genes of ligand–receptor pairs in the ANGPTL, GAS, PDGF, and CXCL signaling pathway networks.

To reveal the mechanism underlying the damage to the iBRB in early DR, we deeply studied the complex communication among Müller cells, endothelial cells, and pericytes. Using CellChat analysis, we found that Müller plays a potential role in the regulation of iBRB through secreting factors (Figure 4A). We found that both groups of Müller cells can secrete VEGF, which can increase vascular permeability and promote the migration and proliferation of vascular endothelial cells and the formation of neovascularization. Vegfr1 (*Flt1*) and Vegfr2 (*Kdr*) receptors are simultaneously expressed on endothelial cells, and Vegf mainly exerts its effect by binding to Vegfr2. Notably, Müller cells themselves also express Vegfr. We speculate that Vegf produced by Müller cells can also act on themselves, but the effect is not clear. However, studies have shown that the Vegf/Vegfr2 signaling pathway is most likely to affect the biological synthesis, secretion, or degradation of brain-derived neurotrophic factor (BDNF) and glial cell-derived neurotrophic factor (GDNF) in Müller cells (Le et al., 2021), promote the survival of Müller cells and have neuroprotective effects. In addition, two groups of Müller cells may act on the receptor Igf2r on Pericyte_B by secreting insulin-like growth factor 2 (Igf2) to regulate its proliferation, differentiation, and survival (Figures 4B,C). Tgf-β is released by Müller cells. It can participate in endothelial cell proliferation, apoptosis, diffusion, and vascular endothelial formation, but its specific role in DR deserves further study.

In DR, abnormal metabolic and pathological conditions, such as oxidative stress and inflammation, can destroy the communication between pericytes and endothelial cells, resulting in the destruction of the BRB. Angiopoietin-like protein 4 (Angptl4) is secreted by endothelial cells and not only acts on Müller cells and pericytes but also affects itself (Figure 4D). Previous studies have confirmed that Angptl4 can regulate the integrity of EC-EC connections, promote an increase in vascular permeability and have a synergistic effect with Vegf (Sodhi et al., 2019). Endothelial cells have a certain regulatory effect on Müller cells and pericytes through Angptl4-Cdh11/Sdc4/Sdc2, but their specific function is worthy of further study (Figure 4E). In addition, we explored some potential methods of pericyte recruitment. Endothelial cells can secrete the protein product Gas6 encoded by growth arrest-specific gene 6, which is an important factor promoting angiogenesis. Gas6 has a selective high affinity for its receptor Axl. Endothelial cells may promote the recruitment and adhesion of pericytes by binding Gas6 to the receptor Axl on pericytes (Lei et al., 2016). Similarly, we found that Pdgfrβ, the receptor of Pdgfβ, was also expressed in the two groups of pericytes. When Pdgfβ secreted by endothelial cells binds to pericyte-specific receptors, it will have the same effect as Gas6. Pdgfβ secreted by endothelial cells binds to the specific receptor Pdgfr of two groups of pericytes, resulting in pericyte recruitment and adhesion to endothelial cells. Interestingly, endothelial cells and Pericyte_B also specifically secrete the chemokines Cxcl12 and Pdgfa, respectively, and act on the corresponding receptors of Pericyte_B and endothelial cells, and whether they have the above recruitment function needs further study.

## Discussion

The iBRB is an important structure that ensures the homeostasis of the retina and plays an important role in the pathogenesis of various retinal diseases. It is important to further study and explore the related mechanisms of the steady state and destruction of the blood–retinal barrier to promote the treatment of diabetic retinopathy. Due to the heterogeneity of the retina and the loss of cell type specificity, high-resolution methods such as scRNA-seq with bulk RNA-Seq approaches have inestimable value for the study of the destruction mechanism of the iBRB in DR. Using scRNA-seq, we identified two different Müller cell subsets and constructed a communication network among endothelial cells, pericytes, and Müller cells in the iBRB.

Müller cells are important cells for maintaining the normal structure and function of the iBRB, especially in cell communication. We defined two new Müller cell subpopulations, *Ctxn3*^+^ Müller and *Ctxn3*^−^ Müller, according to the expression of the *Ctxn3* gene. However, our immunofluorescence experiment results are somewhat ambiguous, and since our results are based on the scRNA-seq, they may just reflect a series of Müller cell phenotypes, so more accurate experiments are needed to verify the correctness of clustering. Our work is consistent with the previous observation that Müller cells participate in the formation and regulation of nerves and blood vessels by secreting cytokines to maintain BRB integrity. Interestingly, there were significant functional differences between the two groups of Müller cells. Our analysis data showed that *Ctxn3*^+^Müller cells specifically overexpressed many genes related to metabolic waste clearance of the BRB, such as aquaporin AQP4 and GABA transporter. However, *Ctxn3*^−^ Müller cells showed upregulation of many genes related to photosensitivity, such as *Pde6b* and *Cngb1*. In addition, compared with *Ctxn3*^−^ Müller cells, *Ctxn3*^+^ Müller cells upregulated the regulation of differentiation inhibitors (Id1-4) and transcription factors Hes1 and Sox9, suggesting that they may have potential roles in promoting endothelial injury and angiogenesis. The above research shows that there is a special Müller cell subpopulation, *Ctxn3*^+^ Müller, which may have important significance in maintaining iBRB and early retinal vascular leakage. It is urgent to reduce the damage of retinal Müller cells to iBRB and thus hinder the progress of DR. Therefore, further research is needed to determine the more specific mechanism of *Ctxn3*^+^ Müller cells in the early stage of DR.

We also investigated the function of endothelial cells in the inner blood–retinal barrier in early DR. This study explored the protective mechanism of endothelial cells and proposed a series of potential early therapeutic targets for DR. The results showed that *Dll4*, *Bmpr2,* and *Nrf2* were highly expressed in endothelial cells. Inhibition of the Dll4-Notch1 pathway promotes excessive sprouts and endothelial proliferation in the retina (Zhu et al., 2022), and the high expression of *Bmpr2* is also conducive to regulating excessive angiogenesis. In an experimental model of *Bmpr2* knockout, vascular endothelial cell proliferation was observed (Theilmann et al., 2020). *Nrf2* can activate the expression of many antioxidant enzymes, and ROS produced by NADPH oxidase can also activate *Nrf2*. Its function in preventing atherosclerosis has been confirmed (Alonso-Pineiro et al., 2021). Since ROS are significantly increased in the early stage of DR, we speculate that *Nrf2* is also involved in endothelial self-protection during DR. Tie2, which plays an important role in vascular stability, is highly expressed in endothelial cells. Previous studies have shown that activated Tie2 increases endothelial cell survival and cell connectivity integrity (Campochiaro and Peters, 2016), thereby stabilizing the vascular system.

After we studied the changes in various cells in DR, our CellChat analysis of the main cells of the iBRB identified the communication and connection modes between them through direct and indirect mechanisms, thus generating important insights into the regulatory mechanism of the iBRB. The cellular communication network is a weighted digraph consisting of significant ligand–receptor pairs between interacting cell groups, showing the number of ligand–receptor interactions detected between different cell groups. The same cell group can send or receive signals. CellChat uses network analysis to infer the strength of different cell groups as senders and receivers of signals during cell communication. Cell grouping is a prerequisite for using CellChat. Before cell communication analysis, cell clustering needs to be carried out carefully to capture cell groups with biological significance. CellChat can quantify the similarity between all significant signal pathways and group them according to the similarity of their cell communication networks. In particular, we observed that the Notch pathway plays an important role in their communication. Previous studies have shown that *Jag1* and *Dll4* are secondary to increased hyperglycemia, activate the normative and non-normative Notch1 pathways, and destroy the endothelial adhesion connection in the retina in diabetes (Miloudi et al., 2019), which is consistent with our findings. Moreover, inhibiting the proliferation of Müller cells by targeting Notch ligands inhibits the overexpression of ECM proteins (Fan et al., 2020) and prevents proliferative diabetes retinopathy and retinal fibrosis. These new findings suggest that the Notch pathway plays an important role in the destruction of the iBRB in the early stage of DR through intercellular communication. We found that in the communication network of the iBRB, Müller cells and endothelial cells are primarily responsible for secreting signal proteins, while pericytes receive secretory factors. Our data suggest that Müller cells regulate the survival of pericytes by secreting Igf2. Moreover, Vegf secreted by Müller cells not only regulates the proliferation of endothelial cells but also acts on Vegfr expressed by itself. Studies have shown that the Vegf/Vegfr2 signaling pathway promotes the survival of Müller cells and has neuroprotective effects (Xu et al., 2019). We found that endothelial cells connect with pericytes through Wnt5b-Fzd4 and affect cell migration and inflammation in angiogenesis through the ncWNT signaling pathway (Zhang et al., 2018; Carvalho et al., 2019), which may be an important target for DR treatment. These newly discovered intercellular communications are crucial to deciphering the regulatory mechanism of the iBRB in the early stage of DR.

In conclusion, we used scRNA-seq to investigate the role and connection of cells in the formation of the iBRB in the early stage of DR, emphasizing the importance of the new Müller subgroup *Ctxn3*^+^ Müller in the mechanism of injury and disease. In addition, our study proposes a global view of new therapeutic targets and cell functions of DR, cell communication, and its relationship in early DR. These data provide new insights into the stability of the iBRB and the mechanism of early destruction in DR. However, our experiment does have the problem of fewer samples, and more experiments and data need to be used to study and prove these findings.

## Materials and methods

### Ethics statement

All animals were housed in a pathogen-free environment and had free access to food. The animal care and use agreement was approved by the Science and Technology Commission of Shanghai Municipality and complies with all applicable institutional and government regulations on the ethical use of animals.

### Animals

The SD rats were fed with high sugar and high fat diet for 4 weeks, and were fasted but watered after 12 h, and administered 40 mg/kg streptozotocin (STZ, 10 g/l, Sigma company, United States) diluted with sterile fresh citrate buffer (0.1 mM, pH 4.3–4.5) to establish the diabetic rat model *via* one-time intraperitoneal injection. If the tail vein blood glucose was higher than 16.7 mmol/l on the third and seventh days after injection, the type 2 diabetes model was successful. If the standard was not met, repeat the injection once. Those who failed to meet the standard twice were excluded. At the same time, the control group used sodium citrate instead of STZ for intraperitoneal injection.

### Tissue processing and cell purification

Animals believed to have diabetes were maintained on a high sugar diet, and tail vein blood samples were regularly collected and measured to assess blood glucose levels. The control group was fed with D12450B 10 kcal% feed, and the experimental group was fed with D12492 60 kcal% feed. The imported feed is produced by American Research Diets. The rats in the experimental group (2 weeks, 4 weeks, 8 weeks) and the control group were anesthetized by intraperitoneal injection and fixed in the supine position. First, the eyeball was removed, and then, the corneoscleral margin was cut. The eyeball was radially incised, the anterior segment and vitreous were removed, and the iris structure was slowly separated from the intact retina and placed in a protective solution.

The tissues were transported in sterile culture dishes with 10 ml of 1x Dulbecco’s phosphate-buffered saline (DPBS; Thermo Fisher, Cat. No. 14190144) on ice. The residual tissue storage solution was removed, and the tissues were then minced on ice. We used 0.25% trypsin (dissociation enzyme; Thermo Fisher, Cat. No. 25200–072) and 10 μg/ml DNase I (Sigma, Cat. no. 11284932001) dissolved in PBS with 5% fetal bovine serum (FBS; Thermo Fisher, Cat. No. SV30087.02) to digest the tissues. The tissues were dissociated at 37°C with a shaking speed of 50 RPM for approximately 40 min. We repeatedly collected the dissociated cells at intervals of 20 min to increase cell yield and viability. The cell suspensions were filtered using a 40 μm nylon cell strainer, and red blood cells were removed by 1X Red Blood Cell Lysis Solution (Thermo Fisher, Cat. No. 00–4,333-57). The dissociated cells were washed with 1x DPBS containing 2% FBS. The cells were stained with 0.4% Trypan Blue (Thermo Fisher, Cat. No. 14190144), and viability was assessed on a Countess® II Automated Cell Counter (Thermo Fisher).

### Preparation and sequencing of the 10x library

Beads with unique molecular identifiers (UMIs) and cell barcodes were loaded close to saturation so that each cell was paired with a bead in gel beads-in-emulsion (GEMs). After exposure to cell lysis buffer, polyadenylated RNA molecules hybridized to the beads. The beads were retrieved into a single tube for reverse transcription. In cDNA synthesis, each cDNA molecule was tagged on the 5′ end (that is, the 3′ end of the messenger RNA transcript) with the UMI and a cell label indicating its cell of origin. Briefly, 10× beads were subjected to second-strand cDNA synthesis, adaptor ligation, and universal amplification. Sequencing libraries were prepared using randomly interrupted whole-transcriptome amplification products to enrich the 3′ ends of the transcripts linked with the cell barcodes and UMIs. All the remaining procedures, including library construction, were performed according to the standard manufacturer’s protocol (CG000206 Rev. D). The sequencing libraries were quantified using a High Sensitivity DNA Chip (Agilent) on a Bioanalyzer 2,100 and with a Qubit High Sensitivity DNA Assay (Thermo Fisher Scientific). The libraries were sequenced on a NovaSeq6000 instrument (Illumina) in 2 × 150 bp mode.

### scRNA-seq analysis

The Read10X() function in the Seurat package (3.2.2) was used to merge the data from all samples into R (4.0.2) and generate an aggregated Seurat object (Butler et al., 2018; Stuart et al., 2019). Low-quality cells (<350 genes/cell, >3,000 genes/cell, <3 cells/gene, >20% mitochondrial genes, and > 20% ribosomal genes) were excluded. Finally, 35,910 single cells were further studied: 11073 normal tissue-derived cells and 24,837 diabetes tissue-derived cells. For identification of cell clusters, the highly variable gene list was first analyzed by principal component analysis. Jackstraw analysis was used to identify important PCs, and the first 20 PCs were used in this process. We used the FindClusters() function to perform clustering (resolution 1.0). We used two data dimensionality reduction algorithms (2D UMAP and tSNE; Becht et al., 2018; Kobak and Berens, 2019) for visualization. For standardized gene expression data, we used the FndAllMarkers function to list the markers of each cell cluster. Compared with other cells, the expression of these marker genes was upregulated by at least 1.3 times. The main cell types were determined according to the markers described in the literature. A cluster identified as red blood cells (cluster 32, cell number 52) was removed, and 10 cell types were finally obtained.

### Gene enrichment analysis

The FindMarkers function was used to identify DEGs between two clusters (adjusted *p* value <0.01 and fold change [FC] >1.3). The R package clusterProfiler (Yu et al., 2012) was used to perform GO (Gene Ontology C, 2015) and Kyoto Encyclopedia of Genes and Genomes (KEGG; Kanehisa et al., 2016) pathway enrichment for the DEGs.

Based on the gene set data for *Rattus norvegicus* in the msigdbr package (selections: C2 for category and CP: KEGG for subcategory; Liberzon et al., 2015), we performed GSVA to analyze the enriched gene sets between different cell subtypes. GSVA mainly converts the expression matrix of genes among different strains into the expression matrix of gene sets among samples to evaluate whether different pathways are enriched among different strains. Basic gene set enrichment uses genes in a predefined gene set to evaluate the distribution trend in the gene table sorted by phenotype correlation, so as to judge its contribution to phenotype. Subsequently, the limma package was used to determine the gene sets with significant differences (Ritchie et al., 2015). Differentially enriched signatures were defined as having FDR adjusted *p* values <0.05 and |mean score difference| values ≥0.1.

### Cell–cell interaction analysis

To visualize and analyze intercellular communications from scRNA-seq data, we conducted CellChat analysis (Jin et al., 2021). We create a new CellChat object from our Seurat object. The cell types were added to the CellChat object as cell metadata. CellChat identified differentially overexpressed ligands and receptors for each cell group and associated each interaction with a probability value to quantify communications between the two cell groups mediated by these signaling genes. Significant interactions were identified on the basis of a statistical test that randomly permuted the group labels of cells and then recalculated the interaction probability.

### Immunostaining

Rats were deeply anesthetized with ketamine and xylazine and then sacrificed by cervical dislocation. The eyes were immediately removed, the corneas were incised, and each eye was immersed in 2% paraformaldehyde (PFA) for 3 h at room temperature. After immersion and fixation, the eyes were washed with phosphate-buffered saline (PBS), and the lenses were carefully removed. The eyes were immersed in 10% sucrose for 30 min, 20% sucrose for 2 h, and 30% sucrose overnight at 4°C for cryoprotection before being embedded in Tissue-Tek optimal cutting temperature compound (OCT) and frozen on dry ice. Eighteen micron sections were cut on a cryostat. The sections were blocked for 1 h with 10% normal goat or donkey serum in PBS with 0.5% Triton X-100, incubated overnight at 4°C with primary antibodies, and then incubated with secondary antibodies.

### SCENIC analysis

SCENIC is a computational method for simultaneous gene regulatory network reconstruction and cell-state identification from single-cell RNA-seq data (Aibar et al., 2017).^1^ We first obtained the scores of different TFs in cell subtypes through R and then screened according to the criteria of avg.exp. ≥ 1, pct.exp. ≥ 20 and RelativeActivity ≥1 to obtain important TFs. We obtained the target gene information of TFs using pyscenic and screened them with the same criteria. The input matrix used for pyscenic was the normalized expression matrix output from Seurat.

According to the expression of TFs and target genes and their importance, we visualized the results using the Cytoscape tool (version 3.8.2; specific screening criteria are shown in the legend). The interactions of each TF and target were merged manually to analyze the overall interactions. We also visualized the target genes of several important TFs with a UMAP plot.

## Data availability statement

The datasets presented in this study can be found in the National Center for Biotechnology Information Gene Expression Omnibus database under accession number GSE209872.

## Ethics statement

The animal study was reviewed and approved by the Science and Technology Commission of Shanghai Municipality.

## Author contributions

YW designed the experiments. YZ performed the data analysis and constructed the datasets. XY drew the figures. SY collected the biopsies and performed the immunofluorescence experiments. XY, QL, and YZ wrote the manuscript. YW, MF, GY, and MC supervised this project. All authors reviewed and edited the manuscript, contributed to the article, and approved the submitted version.

## Conflict of interest

The authors declare that the research was conducted in the absence of any commercial or financial relationships that could be construed as a potential conflict of interest.

## Publisher’s note

All claims expressed in this article are solely those of the authors and do not necessarily represent those of their affiliated organizations, or those of the publisher, the editors and the reviewers. Any product that may be evaluated in this article, or claim that may be made by its manufacturer, is not guaranteed or endorsed by the publisher.

## Supplementary material

The Supplementary material for this article can be found online at: https://www.frontiersin.org/articles/10.3389/fnmol.2022.1048634/full#supplementary-material

## Footnotes

^1^ http://scenic.aertslab.org
