# Supplementary material for: Single-cell RNA sequencing reveals the Müller subtypes and inner blood–retinal barrier regulatory network in early diabetic retinopathy
Source: Front Mol Neurosci. 2022 Dec 1;15:1048634. doi: 10.3389/fnmol.2022.1048634 (PMC9754943; doi:10.3389/fnmol.2022.1048634)
Supplement: Supplementary file 1 [file Data_Sheet_1.doc]

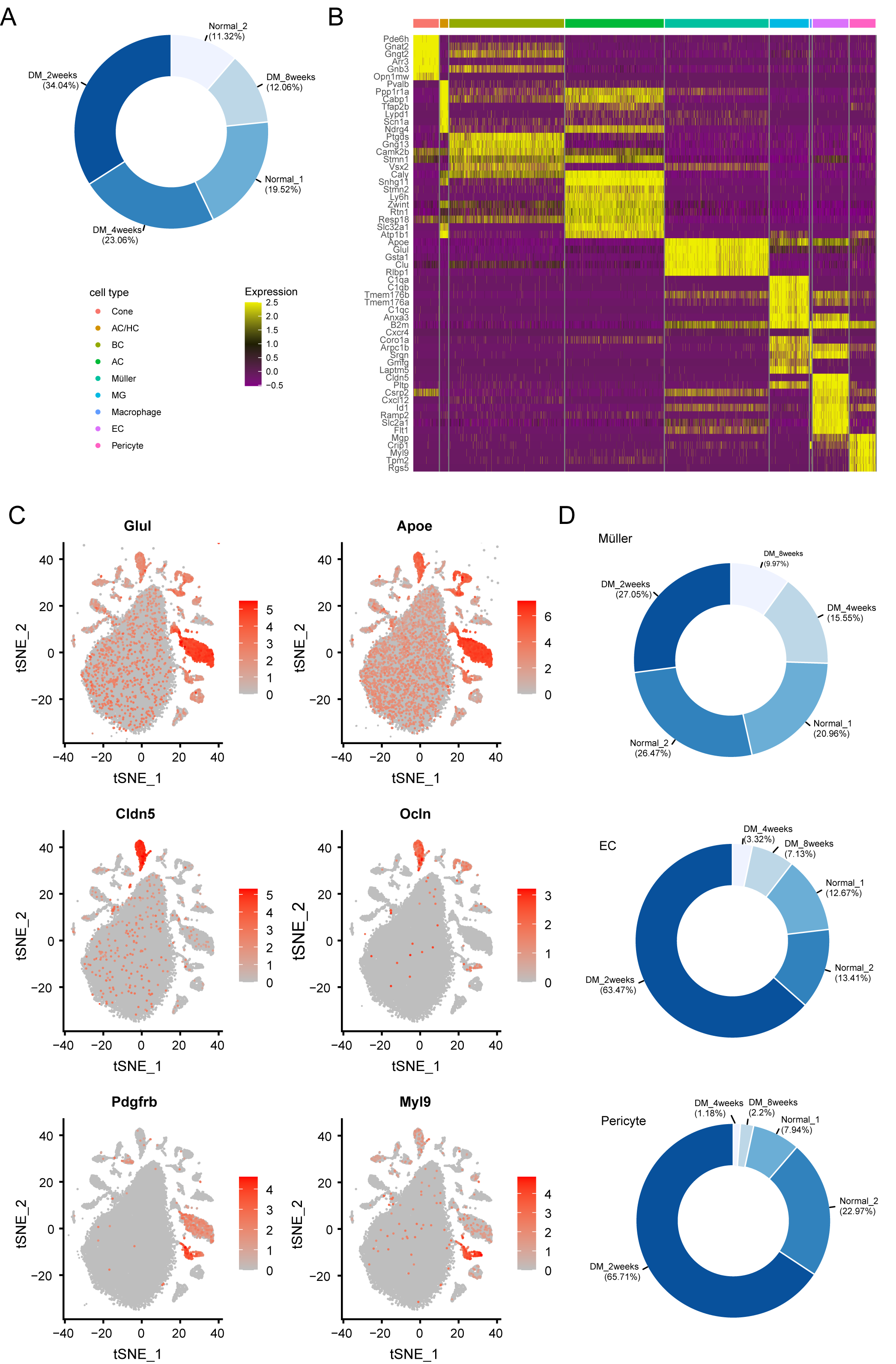


**Figure S1 An atlas of cell types in inner blood retina barrier** (A) The doughnut shows the number and proportion of different samples. (B) The heatmap shows the expression of marker gene in different cell types. (C) Featureplot shows the expression of marker gene in Müller cells, endothelial cells and pericytes. (D) The doughnuts show the number and proportion of different samples in Müller cells, endothelial cells and pericytes.


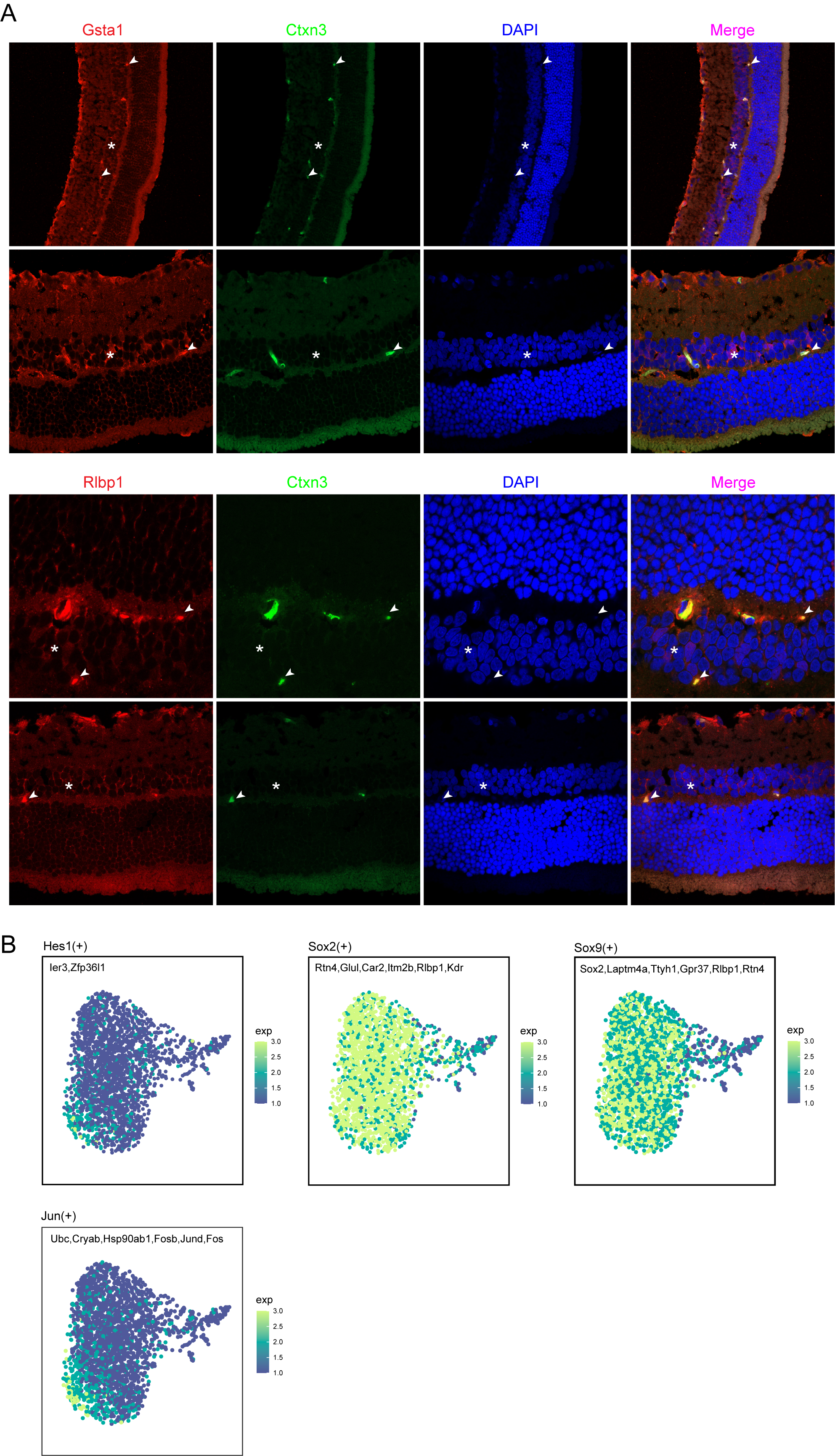


**Figure S2 The characteristics of two subtypes of Müller cells** (A) Immunofluorescence labeling for Gsta1 (red) and Ctxn3 (green) and DAPI nuclear staining (blue) in the rat retina (20×, scale bar 50 µm, first row) (40×, scale bar 20 µm, second row). Immunofluorescence labeling for Rlbp1 (red) and Ctxn3 (green) and DAPI nuclear staining (blue) in the rat retina (63×, scale bar 10 µm, first row) (40×, scale bar 20 µm, second row). *Ctxn3*^+^Müller are indicated by arrowheads, and *Ctxn3*^-^Müller are indicated by asterisk. (B) UMAP showing the expression of target genes of Hes1, Sox2, Sox9 and Jun. The color from dark to light represents increased expression.


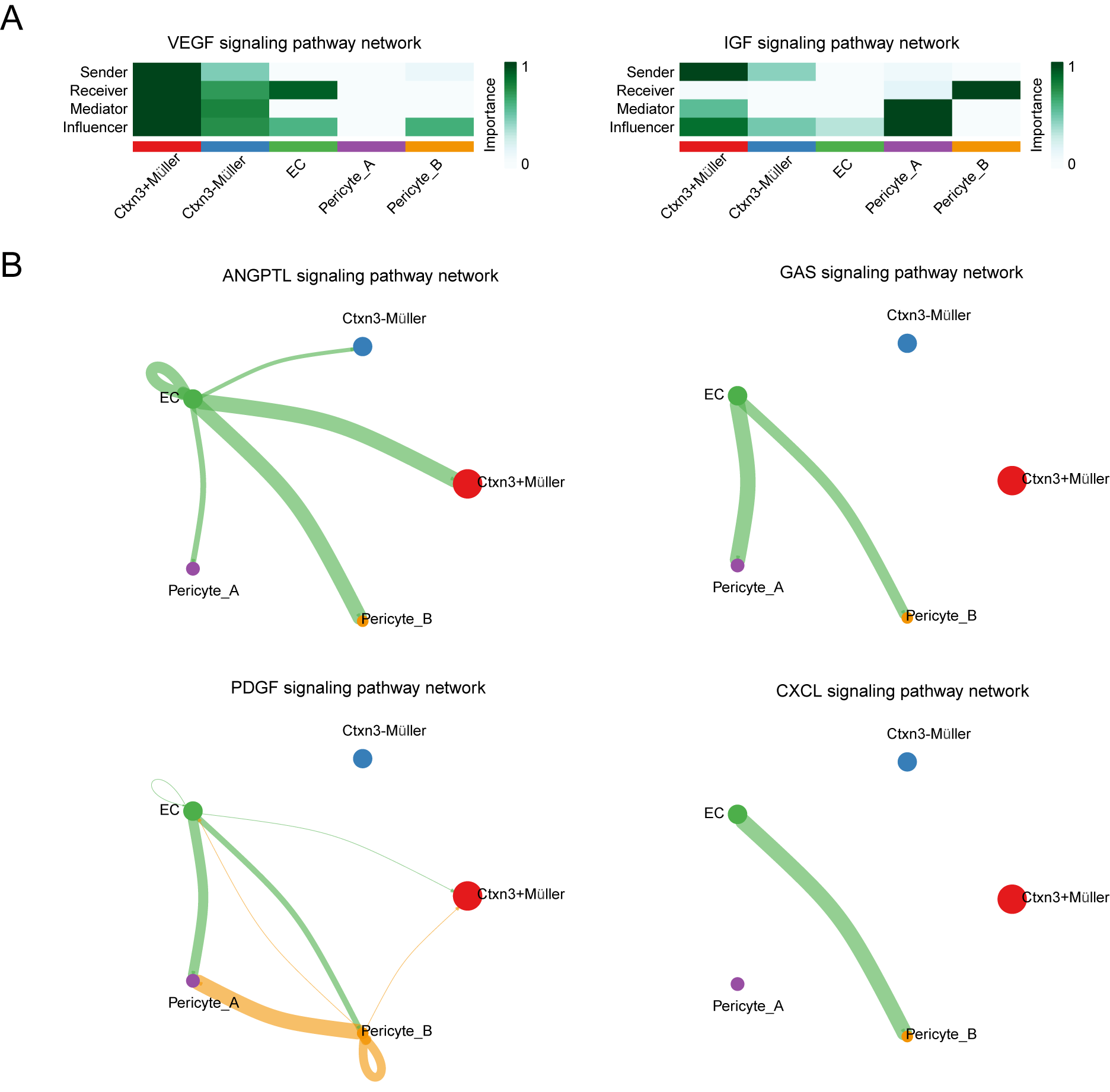


**Figure S3 Communication network between Müller and iBRB** (A) The heatmap shows the sender, receiver, mediator and influencer of VEGF and IGF signaling pathways network. The color represents the strength of the communication signal. (B) ANGPTL, GAS, PDGF and CXCL signaling pathways network between Müller cells, EC and pericytes. The thickness of the line represents the strength of the communication signal.

**Supplementary Table 1 Markers of major cell types**

**Supplementary Table 2 New markers for Müller and iBRB**

| **Cell Types** | **Markers** |
| --- | --- |
| Rod | *Pde6a, Rho, Sag, Gnat1, Nrl* |
| S Cone | *Pde6h, Arr3, Gnat2, Opn1sw, Ccdc136, Ttr* |
| M/L Cone | *Pde6h, Arr3, Gnat2, Opn1mw/Opn1lw, Vopp1, Lmo4* |
| HC | *Pvalb, Lhx1, Pax6, Onecut1* |
| AC | *Snhg11, Pax6, Slc32a1, Crabp1, Nrxn2, Gad1/Gad2, Maf, Tfap2a, Slc6a9, Ebf3* |
| RBC | *Gsg1, Pax6, Slc6a9, Vsx2, Otx2, Prkca, lsl1, Grm6, Cabp5, Vstm2b, Casp7, Rpa1* |
| ON CBC | *Gsg1, Pax6, Slc6a9, Vsx2, Otx2, App, Scgn, Vsx1, Isl1, Grm6* |
| OFF CBC | *Gsg1, Pax6, Slc6a9, Vsx2, Otx2, App, Scgn, Vsx1, Grin2b, Grik1* |
| Müller | *Gsta1, Pax6, Rlbp1, Aqp4, Slc1a3, Apoe, Dkk3, Gpr37, Rax, Hes1, Notch1, Glul* |
| Microglia | *C1qa, Tmem119, Cx3cr1, Apoe, Pax6, P2ry12, Aif1* |
| EC | *Vwf, Pecam1, Cldn5, Cdh5, Tek, Kdr, Flt1* |
| Pericyte | *Rgs5, Kcnj8, Myl9, Cspg4, Pdgfrb, Myh11, Acta2* |
| Macrophage | *Cxcr4, Cd53, Ptprc* |

| **Cell Types** | **Markers** |
| --- | --- |
| *Ctxn3*^+^Müller | *Cd9, Penk, Ctxn3* |
| *Ctxn3*^-^Müller | *Cd9, Penk* |
| EC | *Pltp, Csrp2, Cxcl12, Id1, Ramp2, Slc2a1* |
| Pericyte | *Mgp, Crip1, Tpm2* |

**Supplementary Table 3 Fasted blood glucose levels and weight of animals**

| Group | 15th,November | | 18th,November | | 22th,November | | 29th,November | | 5th,December | | 12th,December | | 26th,December | | 9th,January | |
| --- | --- | --- | --- | --- | --- | --- | --- | --- | --- | --- | --- | --- | --- | --- | --- | --- |
|  | Weight(g) | Blood sugar(mmol/L) | Weight(g) | Blood sugar(mmol/L) | Weight(g) | Blood sugar(mmol/L) | Weight(g) | Blood sugar(mmol/L) | Weight(g) | Blood sugar(mmol/L) | Weight(g) | Blood sugar(mmol/L) | Weight(g) | Blood sugar(mmol/L) | Weight(g) | Blood sugar(mmol/L) |
| Experience group | 542 | 6.6 | 495 | 26.2 | 467 | H1 | 420 | H1 |  |  |  |  |  |  |  |  |
|  | 580 | 7.6 | 518 | 22.2 | 495 | 26.4 | 481 | H1 | 512 | H1 | 530 | H1 |  |  |  |  |
|  | 523 | 6.1 | 447 | 24.2 | 443 | 30.5 | 446 | H1 | 483 | H1 | 471 | H1 | 486 | H1 | 465 | H1 |
| Control group | 507 | 5.8 | 509 | 6.6 | 521 | 5.6 | 532 | 7.4 | 562 | 6.9 | 588 | 10.3 | 602 | 9 | 623 | 10.1 |
|  | 559 | 6.1 | 552 | 6.3 | 581 | 6 | 584 | 7 | 619 | 6.7 | 654 | 9.7 | 678 | 6.6 | 684 | 6.8 |
|  | 534 | 6.3 | 542 | 6.2 | 563 | 5.9 | 587 | 7.5 | 607 | 5.9 | 640 | 8.6 | 656 | 7.9 | 671 | 8.3 |

H1 indicates that the blood glucose instrument is out of range
